# The difference between steroid diabetes mellitus and type 2 diabetes mellitus: a whole-body ^18^F-FDG PET/CT study

**DOI:** 10.1007/s00592-020-01566-w

**Published:** 2020-07-09

**Authors:** Qingqing Zhao, Jinxin Zhou, Yu Pan, Huijun Ju, Liying Zhu, Yang Liu, Yifan Zhang

**Affiliations:** Department of Nuclear Medicine, Ruijin Hospital, Shanghai Jiao Tong University School of Medicine, No. 197, Ruijin 2nd Road, Shanghai, 200025 China

**Keywords:** Steroid diabetes mellitus, Glucocorticoids, Pathogenesis, ^18^F-FDG, PET/CT

## Abstract

**Aims:**

Steroid diabetes mellitus (SDM) is a metabolic syndrome caused by an increase in glucocorticoids, and its pathogenesis is unclear. ^18^F-FDG PET/CT can reflect the glucose metabolism of tissues and organs under living conditions. Here, PET/CT imaging of SDM and type 2 diabetes mellitus (T2DM) rats was used to visualize changes in glucose metabolism in the main glucose metabolizing organs and investigate the pathogenesis of SDM.

**Methods:**

SDM and T2DM rat models were established. During this time, PET/CT imaging was used to measure the %ID/g value of skeletal muscle and liver to evaluate glucose uptake. The pancreatic, skeletal muscle and liver were analyzed by immunohistochemistry.

**Results:**

SDM rats showed increased fasting blood glucose and insulin levels, hyperplasia of islet α and β cells, increased FDG uptake in skeletal muscle accompanied by an up-regulation of PI3Kp85α, IRS-1, and GLUT4, no significant changes in liver uptake, and that glycogen storage in the liver and skeletal muscle increased. T2DM rats showed atrophy of pancreatic islet β cells and decreased insulin levels, significantly reduced FDG uptake and glycogen storage in skeletal muscle and liver.

**Conclusions:**

The pathogenesis of SDM is different from that of T2DM. The increased glucose metabolism of skeletal muscle may be related to the increased compensatory secretion of insulin. Glucocorticoids promote the proliferation of islet α cells and cause an increase in gluconeogenesis in the liver, which may cause increased blood glucose.

## Background

Glucocorticoids are of key clinical use, due to their effective anti-inflammatory, anti-allergic, and immunosuppressive effects [1]. However, excessive glucocorticoids (endocrine corticosteroid secretion or exogenous glucocorticoid intake) in the body often leads to glucose metabolism disorders, a condition termed steroid diabetes mellitus (SDM). The occurrence of SDM seriously affects the survival rate and quality of life of patients [2].

At present, domestic and foreign studies generally classify SDM as type 2 diabetes mellitus (T2DM). It is thought that the occurrence of SDM is related to the damage of islet β cell function, and insulin resistance in major glucose metabolism organs, such as skeletal muscle, liver, and fat caused by glucocorticoids [3, 4]. However, clinical diagnosis and treatment has revealed that SDM patients differ from T2DM patients, and some SDM patients can return to normal blood glucose levels after exposure to excess glucocorticoids. Clinical studies by Giordano et al. [5] demonstrated that the insulin sensitivity index-Matsuda (ISI-Matsuda) and homeostasis model assessment of insulin resistance (HOMA-IR) were not significantly different between Cushing’s Syndrome diabetic and non-diabetic patients. The mechanism of glucocorticoid-induced diabetes requires further investigation.

The previous studies have shown that both acute and chronic glucocorticoids exposure can inhibit insulin release in a dose-dependent manner in rodents [6, 7]. In clinical studies, Van et al. [8] found that after prednisolone treatment, there was an impaired glucose tolerance, reduced C peptide secretion stimulated by arginine, and islet cell dysfunction in healthy volunteers. However, some studies reported that islet β cell function and mass increased following glucocorticoid treatment, due to the body’s compensatory effect [9, 10]. Skeletal muscle is the main organ involved in insulin-mediated glucose uptake (> 80%). Studies show that glucocorticoids can directly interfere with insulin signaling in skeletal muscle cells, inhibit glucose transporter 4 (GLUT4) expression, and reduce insulin-stimulated glucose uptake and glycogen synthesis in isolated skeletal muscle [11, 12]. However, some studies have shown that after dexamethasone treatment in rats, the expression of GLUT4 in skeletal muscle does not decrease, and the glycogen content in skeletal muscle and liver increases [13]. Studies have shown that glucocorticoids also seem to cause insulin resistance by promoting liver gluconeogenesis and adipogenesis [14].

Taken together, current research on the pathogenesis of SDM is limited to in vitro conditions or the assessment of overall glucose metabolism, and neither can reflect the glucose metabolism of tissues and organs under living conditions. Due to ^18^F-FDG having similar biochemical properties to glucose, it can enter cells through the glucose transporter on the cell membrane, where it is phosphorylated to 6-PO_4_-^18^F-FDG by hexokinase, and it remains in the cell. ^18^F-FDG PET/CT imaging can reflect glucose metabolism of tissues and organs in the body [15, 16]. At present, ^18^F-FDG PET imaging has been used to study the glucose metabolism of organs such as the liver, fat, skeletal muscle, and myocardium [17–19], and the results show that this approach allows for the effective evaluation of glucose uptake by these organs.

The aim of this study was to investigate the pathogenesis of SDM and how it differs from T2DM, through the establishment of SDM [20] and T2DM rat models and subsequent ^18^F-FDG micro-PET/CT imaging and immunohistochemical analyses.

## Materials and methods

### Animals care

The experimental rats were purchased from Shanghai SLAC Laboratory Animal Co., Ltd. (Shanghai, China), and housed in the laboratory animal center of Ruijin hospital, which is affiliated to the medical college of Shanghai Jiao Tong University. The temperature was maintained between 22 and 24 °C, and rats were subjected to a 12/12 h light/dark cycle (lights on at 07:00 am) and provided food (Shanghai SLAC company, China) and water ad libitum. The animal study was approved by the Ethics Committee of Shanghai Jiao Tong University School of Medicine (Shanghai, China) and was conducted in accordance with the ethical principles governing animal welfare, rearing, and experimentation.

### Dexamethasone treatment and experimental method

The basal body weight and fasting blood glucose (FBG) values of 35 male Wistar rats (250–300 g) were measured after 1 week of adaptive feeding. The experimental rats were randomly divided into 2 groups, with 20 in the dexamethasone treatment group (SDM) and 15 in the control group (CTL). SDM rats were injected intraperitoneally with dexamethasone (Sigma-Aldrich, USA) (10 mg/kg) daily, and 5 were used for orbital blood collection, 5 for glucose tolerance tests, and 10 for PET/CT imaging. CTL rats were intraperitoneally injected with a corresponding dose of normal saline daily, and 5 were used for orbital blood collection, 5 for glucose tolerance tests, and 5 for PET/CT imaging. In the SDM and CTL groups, changes in body weight and FBG were monitored every other day. Orbital blood collection, glucose tolerance tests, and PET imaging were performed on days 0, 3, 7, 11, and 15 of the experimental time frame.

GK rat is a spontaneous type 2 diabetic rat model, derived from the Wistar rat strain. The basal body weight and FBG values of 10 male GK rats (100–150 g), were measured after 1 week of adaptive feeding with ordinary feed, and the first imaging was performed. High-fat diets were used to accelerate their disease progression, and changes in body weight and blood glucose were monitored once a week, and orbital blood collection and small animal imaging was performed every 1–2 months.

### FBG, serum insulin, and intraperitoneal glucose tolerance test

After 12 h fasting, blood was drawn from the tail tip of the rats, and their FBG was measured using a Bayan automatic blood glucose meter (Contour TM TS, Bayer, Germany). Similarly, after overnight fasting, blood was collected from the orbits of the rats, under anesthesia. The blood was centrifuged at 2000×*g* at 4 °C for 20 min, before the supernatant was collected and stored at − 80 °C. Serum insulin was detected with a Rat Insulin -ELISA Kits (Crystal Chem, USA). CTL, SDM, T2DM rats were euthanized after the last orbital blood sampling, and the tissues were dissected for RNA and immunohistochemistry.

For the intraperitoneal glucose tolerance test, after overnight fasting, the rats were given a glucose load (1 g/kg) by intraperitoneal injection of a 50% glucose solution. The blood glucose was measured as FBG at 0, 30, 60, 90, and 120 min following glucose loading. For statistical analyses, the blood glucose change curves were plotted and the area under the curve (AUC) calculated [21].

### Micro-PET/CT imaging

After 12 h overnight fasting, rats were anesthetized with 3% pentobarbital sodium (Sigma-Aldrich, USA) (30 mg/kg) by intraperitoneal injection. The tail vein was injected with ^18^F-FDG at a dose of approximately 7.4 MBq. After 30 min, rats were fixed in the prone position on the center of a micro-PET/CT (Inveon MM Platform, Siemens Preclinical Solutions, Knoxville, Tennessee, USA) scan bed field of view (FOV) and scanned under anesthesia. The PET/CT equipment has a resolution of 1.5 mm, an aperture of 5.7 cm, and an axial FOV of 8.5 cm. The micro-PET/CT equipment acquisition workstation was Inveon Acquisition Workplace (IAW) 1.5.0.28. A new workflow was established before data acquisition, including CT Acquisition, Reconstruction, PET Acquisition, PET Histogram, and PET Reconstruction. The static scan data were collected under the conditions of 80 kV voltage, 500 μA current, and 1100 ms exposure for 10 min, and then PET data were collected. The collected data were reconstructed with IAW software through attenuation correction, and the three-dimensional ordered subsets maximization algorithm (OSEM3D) was used to reconstruct the coronal, transverse, and sagittal tomographic images for analysis. The reconstructed images were obtained using Siemens Inveon Research Workplace (IRW) 3.0 to obtain 3D Regions of Interest (ROI). In this study, the upper limb epitrochlearis muscle [22], the upper right portion of the liver, and the myocardial apex of heart were measured to represent each organ’s glucose metabolism, respectively. Finally, the %ID/g max value of the ROI was obtained for quantitative analysis [23].

### Quantitative real-time PCR (q RT-PCR)

RNA extraction and cDNA synthesis from muscle were performed using Trizol reagent (Thermo Fisher Scientific, Waltham, MA, USA) and PrimeScript™ RT Reagent Kit with gDNA Eraser (Takara, China Da Lian), respectively, according to the manufacturers’ protocols. Total RNA (500 ng) was amplified on a StepOne Fast Real-Time PCR System (Thermo Fisher Scientific), using TB Green™ Premix Ex Taq™ II (Tli RNaseH Plus) (Takara, China Da Lian) for real-time PCR after cDNA synthesis. The standard curve for quantification was derived as per a modified version of a previously described method. Fold change of the gene expression was calculated by 2^−ΔCt^ relative to the internal reference gene (glyceraldehyde3-phosphate dehydrogenase, GAPDH). The primer sequences used are as follows: GLUT4: forward 5′-GGGCTGTGAGTGAGTGCTTTC-3′, reverse 5′-CAGCGAGGCAAGGCTAGA-3′; insulin receptor substrate 1 (IRS-1): forward 5′-ATGTGGAAATGGCTCGGA-3′, reverse 5′-TAAGGCAGCAAAGGGTAGGC-3′; Phosphatidylinositol 3-kinase (PI3K)-p85α: forward 5′-TTAAACGCGAAGGCAACGA-3′, reverse 5′-CAGTCTCCTCCTGCTGTCGAT-3′;GAPDH: forward 5′-AGGTCGGTGTGAACGGATTTG-3′, reverse 5′-TGTAGACCATGTAGTTGAGGTCA-3′.

### Histological analyses

After the rats were euthanized, the excised pancreatic tissue and epitrochlearis muscle of the upper limbs were immersed in 4% paraformaldehyde (Sigma-Aldrich, USA) for 48 h. Tissues were washed in 70% ethanol, embedded in paraffin, and sectioned onto glass slides. The slides were dewaxed with xylene, dehydrated with ethanol, and washed with PBS. The slides were then incubated in a hydrogen peroxide blocking solution for 10 min at 18–25 °C to block endogenous peroxidase activity. Anti-masking/epitope retrieval of the antigen was performed by high-pressure heating of 1 mMol Tris–EDTA (pH 9.0). Slides were incubated in a protein blocking solution (Sigma-Aldrich, USA), before the pancreatic tissue was tested for insulin (1:100, ab7842, Abcam, CA, USA) and glucagon (1:200, ab10988, Abcam, CA, USA). Skeletal muscle was tested for GLUT4 (1:500, ab654, Abcam, CA, USA), IRS-1 (1:100, ab52167, Abcam, CA, USA), and PI3Kp85α (1:100, ab182651, Abcam, CA, USA). After overnight incubation at 4 °C, the slides were immunostained with secondary antibodies from EnVision reagent (HRP/rabbit and mouse, Dako, K5007 kit, Denmark). DAB was added and allowed to develop under microscope observation until brown staining was visible. Slides were counterstained with hematoxylin (Sigma-Aldrich, USA). Representative slides of glucagon and insulin were used to quantify the mass and islet area of islet α and β cells.

### Statistical analyses

Results are presented as mean ± standard error of the mean (unless otherwise stated). Statistical analyses were performed using GraphPad Prism version 7.0 (GraphPad Software, Inc., La Jolla, CA, USA). Data sets were analyzed for statistical significance using a two-tailed unpaired Student t test. *P < 0.05; **P < 0.01; ***P < 0.001; ****P < 0.0001.

## Results

### Metabolic changes in SDM and T2DM rats

**Fig. 1:**
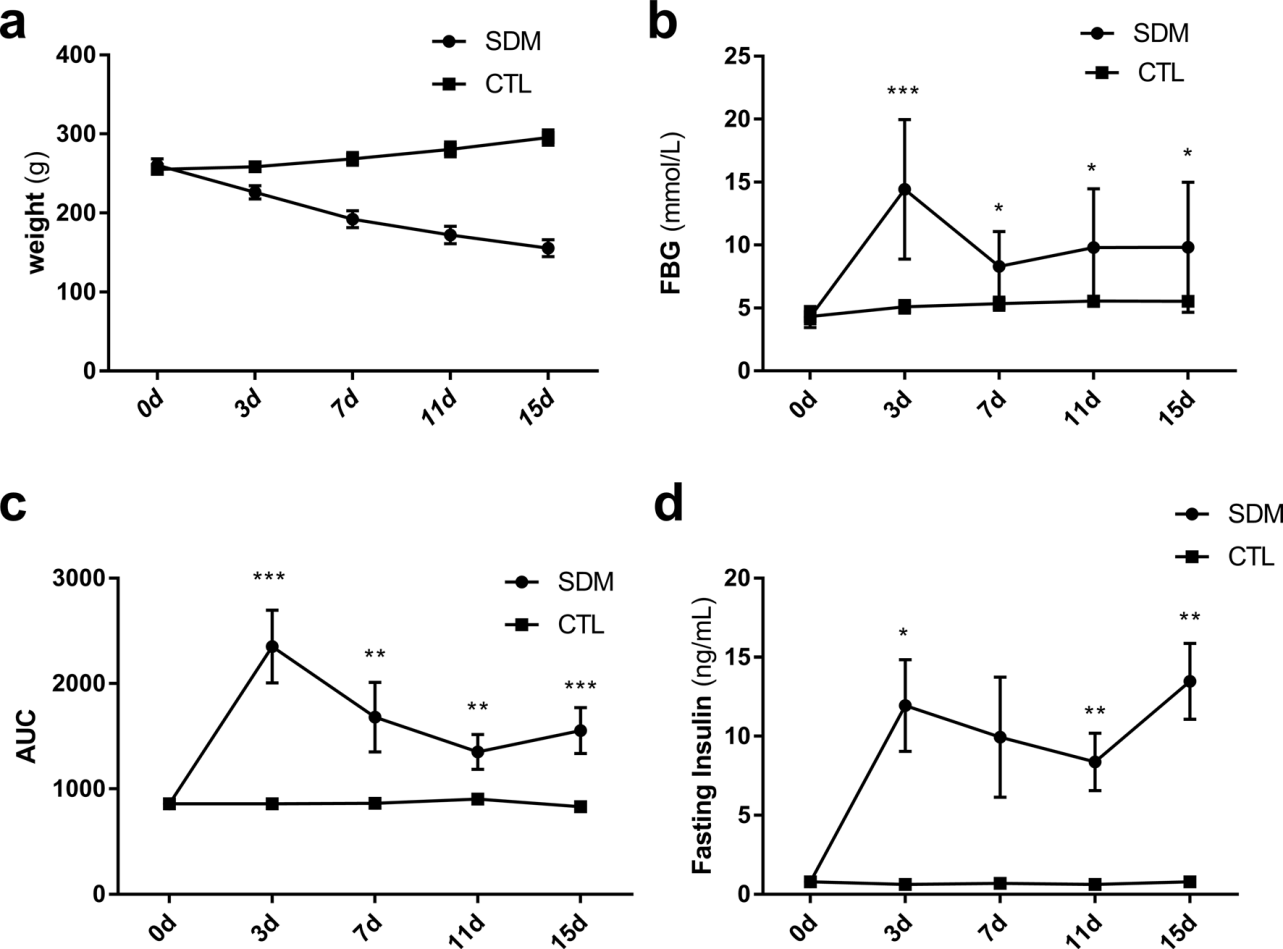
Metabolic changes in SDM rats (weight, FBG, AUC, and fasting insulin). **a** Rat weight over time (SDM, *n* = 10; CTL, *n* = 7); **b** FBG over time (SDM, *n* = 10; CTL, *n* = 7); **c** AUC for the glucose tolerance test over time (*n* = 4); **d** Fasting insulin over time (*n* = 4). *Compared to CTL rats

After dexamethasone treatment in SDM rats, their weight gradually decreased over time (Fig. 1a). Studies have shown that different doses of dexamethasone intervention can cause weight loss in rats [24, 25]. FBG dropped after a transient rise on the third day of administration, and slowly increased to 9.8 mmol/L from day 7 to 15, which was significantly higher than that of the CTL rats (Fig. 1b). Glucose tolerance test results show the AUC of SDM rats is significantly higher than that of the CTL rats (Fig. 1c). SDM rats showed significant hyperinsulinemia after dexamethasone injection, which were significantly higher than the CTL rats (Fig. 1d).

**Fig. 2:**
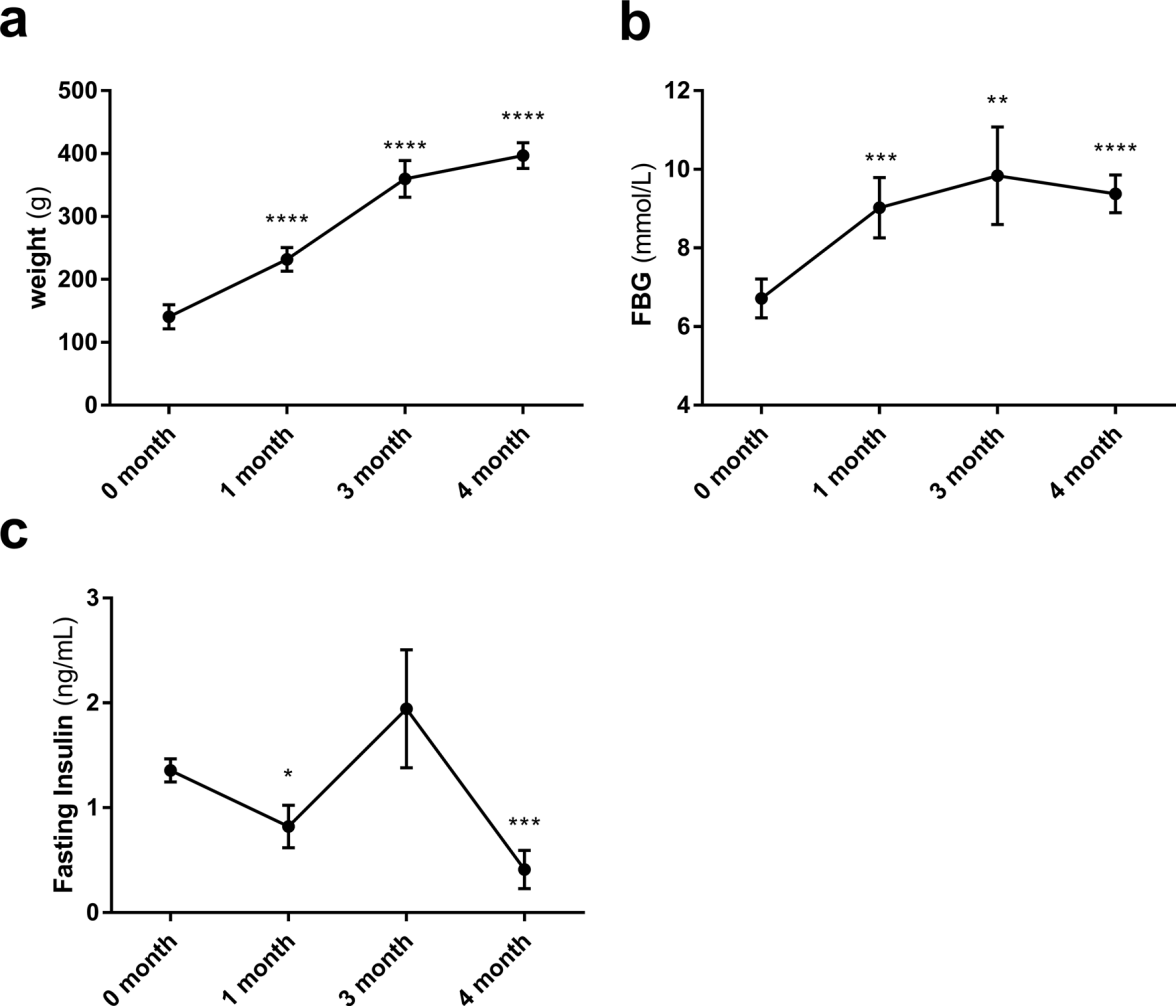
Metabolic changes in T2DM rats (weight, FBG, and fasting insulin). **a** T2DM rat weight over time (*n* = 6); **b** T2DM rat FBG over time (*n* = 4); **c** T2DM rat fasting insulin over time (*n* = 4). *Compared to month 0

The modeling time of T2DM model lasted 4 months, and the FBG values (high-fat diet 4 months) matched that of the SDM rats (day 15). The weight of the T2DM rats gradually increased over disease progression (Fig. 2a). FBG gradually increased with time, before stabilizing (about 9.4 mmol/L) (Fig. 2b). Fasting insulin showed a decreasing trend, and there was a significant difference between 0 and 4 months (Fig. 2c).

### Glucose uptake changes in skeletal muscle and liver of SDM and T2DM rats

**Fig. 3:**
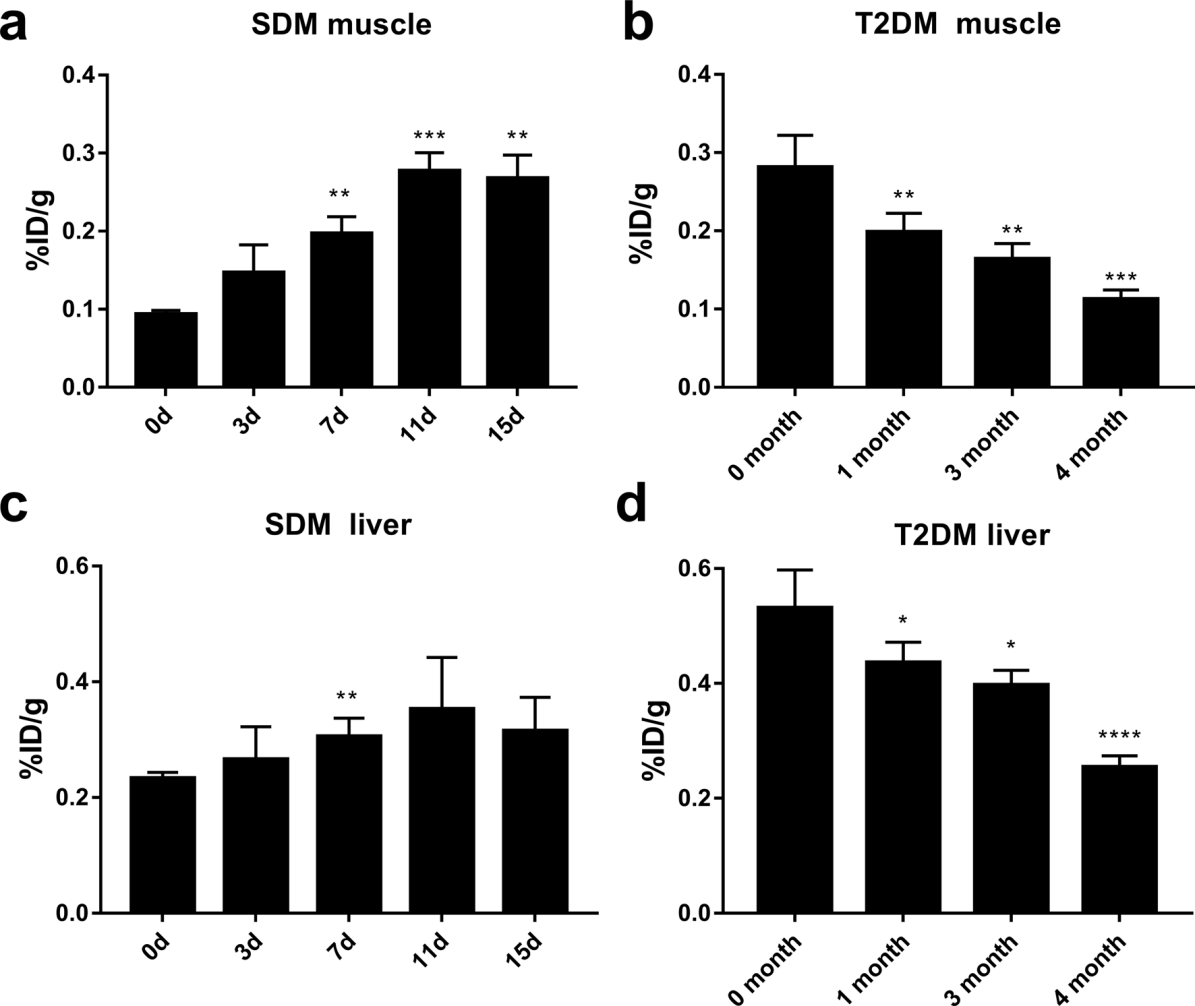
Glucose uptake (% ID/g max) of skeletal muscle and liver in PET/CT imaging over time. **a** glucose uptake by skeletal muscle in SDM rats; **b** glucose uptake by skeletal muscle in T2DM rats; **c** glucose uptake by the liver in SDM rats; **d** glucose uptake by the liver in T2DM rats. *n* = 4, *Indicates that the statistical analysis of SDM rats is compared to day 0, and that of T2DM rats is compared to month 0

There was no difference of FBG between the PET/CT imaged rats and non-imaged ones during the study period in both the SDM and T2DM groups, respectively. The mass of ROI in skeletal muscle was 94.3 (62.7 to 117.6) mg, the mass of ROI in liver was 413.4 (351.6 to 477.2) mg (median and range values). The obtained  %ID/g max value was corrected for mass. ^18^F-FDG PET/CT imaging results showed that the glucose uptake by skeletal muscle in SDM rats gradually increased over time; the %ID/g of glucose uptake in skeletal muscle on days 7 (*P *< 0.01), 11 (*P *< 0.001), and 15 (*P *< 0.01) is significantly higher than at day 0 (Fig. 3a). The glucose uptake by skeletal muscle in T2DM rats gradually decreased over time, and there was a statistical difference between months 1 (*P *< 0.01), 3 (*P *< 0.01), and 4 (*P *< 0.001) compared to 0 months (Fig. 3b). SDM liver glucose uptake did not change significantly over time. The increase in liver glucose uptake on day 7 was significantly different to day 0 (*P *< 0.01) (Fig. 3c). T2DM liver glucose uptake gradually decreased, and it was significant at months 1 (*P *< 0.05), 3(*P *< 0.05), and 4 (*P *< 0.0001) compared to 0 months (Fig. 3d). The overall level of liver glucose uptake in SDM rats during disease progression was lower than that in T2DM rats (*P *< 0.01).

Meanwhile, myocardial glucose metabolism was also measured. The mass of ROI in the myocardium was about 50.9 (36.3 to 58.3) mg (median and range values). No changes in myocardial glucose metabolism were found in SDM rats during the study, while the myocardial glucose uptake was reduced in T2DM rats at months 4 (*P *< 0.001). The current study is focused on the glucose metabolism in major organs of skeletal muscle and liver, and the heart is not expected to contribute to the total body glucose metabolism significantly, as the mass of the heart muscle is much lower than the body skeletal muscle.

### Immunohistochemical analysis of skeletal muscle and liver in rats

**Fig. 4:**
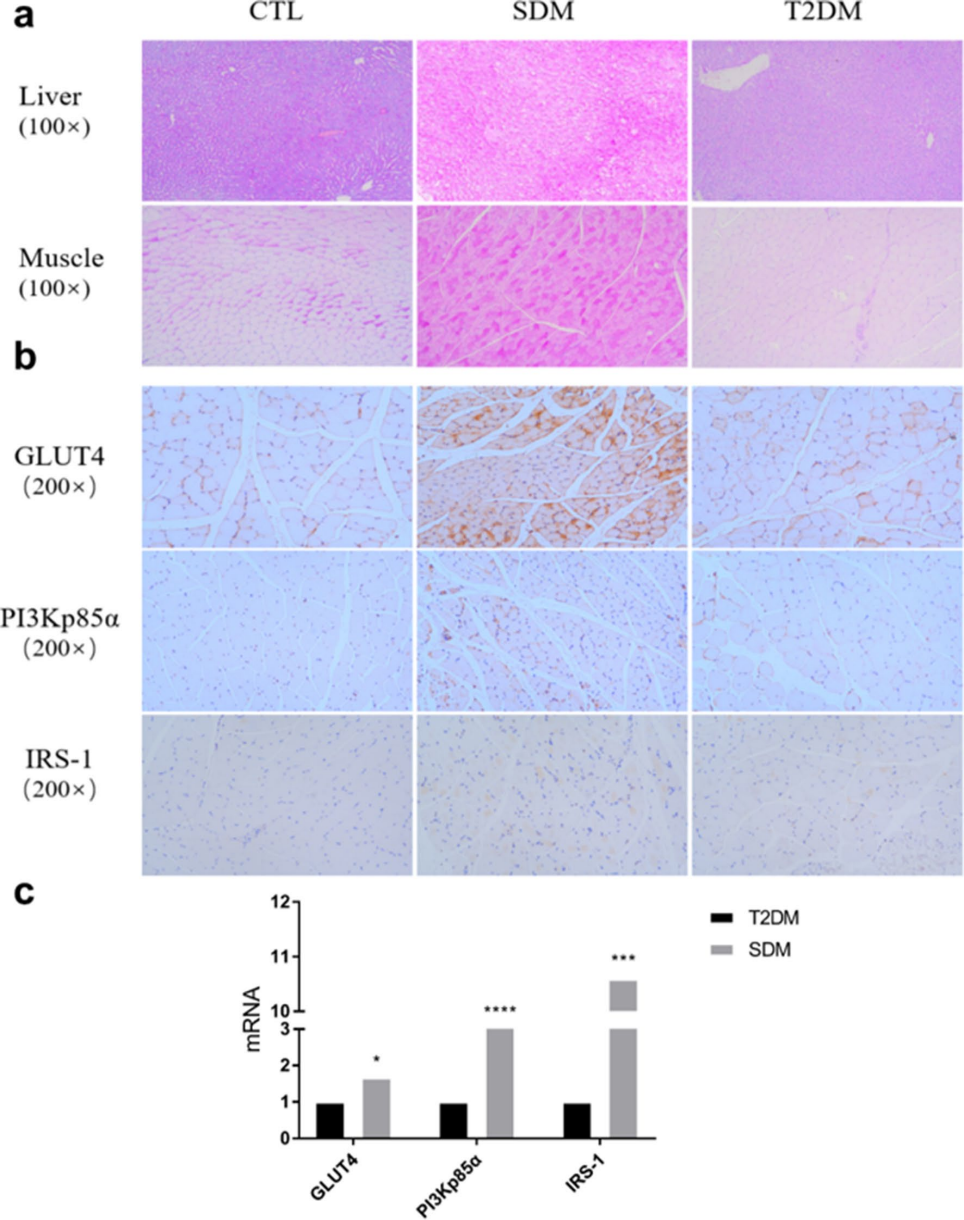
Immunohistochemistry and RNA analysis of skeletal muscle and liver in rats. **a** analysis of glycogen content (PAS) in the liver and skeletal muscle of CTL, SDM, and T2DM rats; **b** immunohistochemical analysis of GLUT4, PI3Kp85α, and IRS-1 in the skeletal muscle of CTL, SDM, and T2DM rats; **c** analysis of RNA expression levels of GLUT4, PI3Kp85α, and IRS-1 genes in the skeletal muscle of SDM and T2DM rats. *n* = 4, *Compared to T2DM rats

Immunohistochemical analysis of skeletal muscle and liver in SDM rats (15 days) and T2DM rats (4 months) showed that SDM rats had higher glycogen levels in the liver and skeletal muscle than CTL and T2DM rats (Fig. 4a).GLUT4, IRS-1, and PI3Kp85α were higher in the skeletal muscle of SDM rats than that of CTL and T2DM rats; there was no significant difference in the expression of GLUT4, IRS-1, and PI3Kp85α in skeletal muscle between T2DM and CTL rats (Fig. 4b). Although GAPDH may change in diabetes as reported in some studies [26], there is no difference in GAPDH expression between SDM and T2DM in the current study. Therefore, the mRNA data of SDM and T2DM rats were statistically analyzed. The results showed that the levels of GLUT4 (*P *< 0.05), IRS-1 (*P *< 0.0001), and PI3Kp85α (*P *< 0.001) mRNA in the skeletal muscle of SDM rats were significantly higher than those of T2DM rats (Fig. 4c).

### Immunohistochemical analysis of pancreatic in rats

**Fig. 5:**
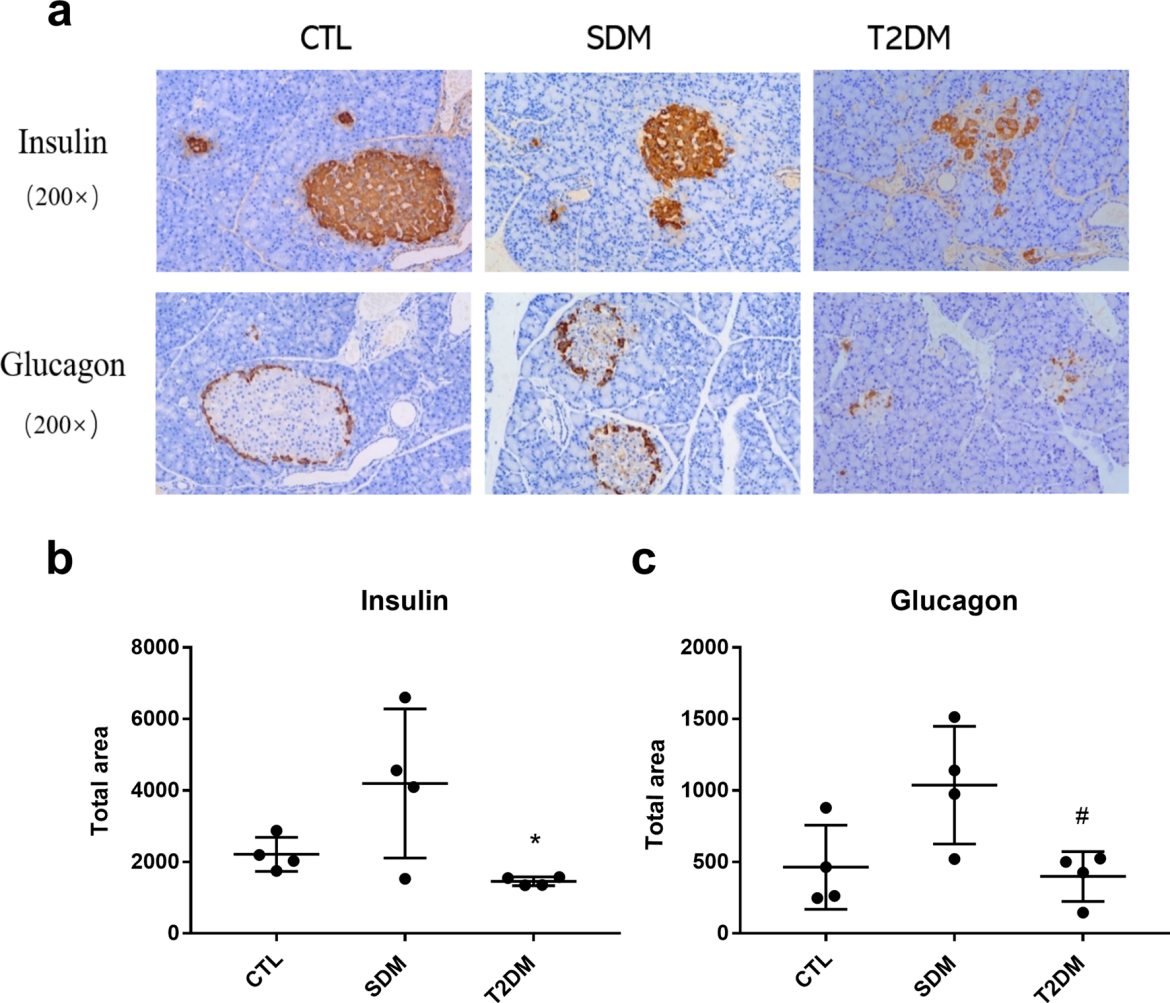
Immunohistochemical analysis of pancreatic in rats. Immunohistochemical analysis of insulin and glucagon in pancreatic of CTL, SDM, and T2DM rats (*n* = 4). *Compared to CTL rats; #compared to SDM rats

Immunohistochemical analysis of pancreatic insulin and glucagon in SDM rats (15 days) and T2DM rats (4 months) (Fig. 5) showed that the total area of pancreatic islet β cells in SDM rats increased, but it was not significantly different to the CTL rats (*P *= 0.19). The total area of pancreatic β cells in T2DM rats was significantly lower than that in the CTL group (*P *< 0.05), and there was no significant difference compared to SDM rats (*P *= 0.06). The total area of islet α cells in SDM rats increased; while there was no significant difference compared to CTL rats (*P *= 0.10), there was compared to T2DM rats (*P *< 0.05). The total area of islet α cells in T2DM rats was not significantly different to CTL rats (*P *= 0.72).

## Discussion

This is the first study to report on the differences in glucose uptake in skeletal muscle and the liver during the progression of SDM and T2DM using a molecular imaging approach. The study found that with the same level of hyperglycemia, the fasting insulin levels in SDM rats was significantly increased, as was pancreatic β cell proliferation. T2DM rats were observed to have lower fasting insulin levels, and the total area of islet β cells decreased. Glucose uptake in the skeletal muscle of SDM rats increased, which was accompanied by an up-regulation of PI3 Kp85α, IRS-1, and GLUT4; the glycogen content of the liver and skeletal muscle also increased in SDM rats. Glucose uptake and glycogen content in T2DM rats’ skeletal muscle and liver decreased.

Pancreatic β cells play a vital role in glucose metabolism. In vitro studies show that glucocorticoids can reduce β cell glucose uptake and phosphorylation, thereby reducing ATP synthesis and Ca^+^ influx, leading to reduced insulin biosynthesis and release [6, 27]. In addition, glucocorticoids can reduce β cell mass by inducing apoptosis [28, 29]. In contrast, a study by Rafacho et al. [30] showed that with high-dose dexamethasone treatment, rat pancreatic islet β cells underwent adaptive changes, the ability of the islets to respond to glucose increased, and insulin compensated secretion increased. In this study, unlike T2DM, SDM manifested as islet β cell compensatory hyperplasia and increased insulin secretion, indicating that changes in islet β cell mass are not the main cause of SDM. However, it is interesting to note that pancreatic α cell hyperplasia was also observed in this study. Previous studies have shown that glucocorticoids can cause pancreatic α-cell proliferation and induce hyperglucagonemia, leading to increased blood glucose and β cell generation compensatory hyperplasia [31, 32]. Glucocorticoid-induced α cell proliferation may be one of the causes of SDM. In addition, glucocorticoids may affect the release of other hormones in the pancreas, such as somatostatin, amylin, and ghrelin, which may also be a cause of glucocorticoid-induced diabetes [33].

Skeletal muscles represent the predominant peripheral site of insulin-dependent glucose disposal [6]. In skeletal muscle, after insulin binds to its receptor, it activates IRS-1, which in turn activates downstream PI3 Kp85α. Protein kinase B (PKB) is subsequently activated by phosphorylation. Once activated, GLUT4 translocation from intracellular vesicles to the cell membrane is enhanced, glucose uptake increased, and glycogen synthesis increased [13]. Studies show that glucocorticoids can induce insulin resistance by directly interfering with insulin signaling in skeletal muscle [34]. Burén et al. [35] found that dexamethasone treatment can reduce insulin-stimulated glucose uptake in rat muscles without reducing basal glucose uptake, and GLUT4 expression increased. Ruzzin et a1. [13] also found that dexamethasone caused insulin resistance in rat skeletal muscle, but the content of glycogen in skeletal muscle and liver increased. Similarly, in this study, an increase in GLUT4 expression, glucose uptake and the glycogen content in skeletal muscle was observed, suggesting that increased insulin compensated for skeletal muscle insulin resistance caused by glucocorticoids [36]. In T2DM rats, lower insulin levels and insulin resistance may be an important cause of reduced glucose uptake in skeletal muscle [37, 38]. Here, it was found that under the same blood glucose levels, compared to T2DM, SDM rats skeletal muscle had a stronger capacity for glucose uptake and utilization. The change in glucose uptake of skeletal muscle is not the main reason for the increase in blood glucose in SDM.

The liver is the main organ of gluconeogenesis and plays an important role in glucose metabolism. In the state of starvation or fasting, gluconeogenesis in the liver increases, and glycogen is broken down and phosphorylated into glucose-6-phosphate, which is then converted into glucose and released into the blood under the action of glucose-6-phosphatase. When the blood glucose rises, it will not only competitively inhibit the uptake of ^18^F-FDG by liver cells, but also feedback inhibit the activity of glucose-6-phosphatase in liver cells; 6-phosphate-FDG cannot rapidly release the phosphate to release the glucose into the blood, and thus it accumulates in the liver [39]. In the present study, the initial increase in T2DM rat blood glucose coincided with a significant increase in liver ^18^F-FDG uptake. However, during the development of T2DM disease, with the occurrence of insulin resistance, the decrease of insulin levels, the development of fatty liver, the liver ^18^F-FDG gradually decreased [40]. In SDM rats, although their blood glucose was also high, as glucocorticoids can promote liver gluconeogenesis, the rate-limiting enzyme phosphoenolpyruvate carboxy-kinase and glucose-6-phosphatase activity increased, leading to increased liver glucose output [41, 42], and a liver ^18^F-FDG uptake lower than in T2DM rats. The uptake of glucose in the liver by glucose transporter 2 is independent of insulin, and the effect of insulin on ^18^F-FDG uptake in the liver of SDM rats is not significant [43]. Taken together, unlike T2DM, the increased glucose output of the liver plays a more important role in the increase of blood glucose in SDM.

As a molecular imaging detection instrument, PET/CT can reflect the metabolic status of tissues and organs under living conditions. In this study, one limitation was that a glucose clamp test was not performed during PET/CT imaging. However, our study conducted a parallel control study with T2DM rats and found that SDM rats have comparatively increased skeletal muscle glucose uptake and lower liver glucose uptake, suggesting that the main reason for the increase in blood glucose in SDM may be the increase in liver gluconeogenesis and increased glucose output.

In conclusion, our data indicate that the increased glucose metabolism of skeletal muscle in SDM rats may be related to the increased compensatory secretion of insulin. The pathogenesis of SDM is mainly related to the islet α cell hyperplasia and the increase of gluconeogenesis in the liver caused by Glucocorticoids. These findings provide new insight into the pathogenesis of SDM, which is useful in the development of novel treatment strategies.
